# Self-Reported Mobile Health-Based Risk Factor and CHA_2_DS_2_-VASc-Score Assessment in Patients With Atrial Fibrillation: TeleCheck-AF Results

**DOI:** 10.3389/fcvm.2021.757587

**Published:** 2022-01-19

**Authors:** Astrid N. L. Hermans, Monika Gawałko, Henrike A. K. Hillmann, Afzal Sohaib, Rachel M. J. van der Velden, Konstanze Betz, Dominique Verhaert, Daniel Scherr, Julia Meier, Arian Sultan, Daniel Steven, Elena Terentieva, Ron Pisters, Martin Hemels, Leonard Voorhout, Piotr Lodziński, Bartosz Krzowski, Dhiraj Gupta, Nikola Kozhuharov, Henri Gruwez, Kevin Vernooy, Nikki A. H. A. Pluymaekers, Jeroen M. Hendriks, Martin Manninger, David Duncker, Dominik Linz

**Affiliations:** ^1^Department of Cardiology, Maastricht University Medical Center and Cardiovascular Research Institute Maastricht, Maastricht, Netherlands; ^2^Institute of Pharmacology, West German Heart and Vascular Center, University Duisburg-Essen, Essen, Germany; ^3^1st Department of Cardiology, Medical University of Warsaw, Warsaw, Poland; ^4^Hannover Heart Rhythm Center, Department of Cardiology and Angiology, Hannover Medical School, Hannover, Germany; ^5^Barts Heart Center, St Bartholomew's Hospital, London, United Kingdom; ^6^Department of Cardiology, King George Hospital, Ilford, United Kingdom; ^7^Department of Cardiology, Radboud University Medical Center, Nijmegen, Netherlands; ^8^Department of Cardiology, University Clinic of Medicine, Medical University of Graz, Graz, Austria; ^9^Department of Electrophysiology, University of Cologne, Heart Center, Cologne, Germany; ^10^Department of Cardiology, Rijnstate Hospital, Arnhem, Netherlands; ^11^Liverpool Heart and Chest Hospital, Liverpool, United Kingdom; ^12^Department of Cardiology and Cardiovascular Research Institute Basel, University Hospital Basel, University of Basel, Basel, Switzerland; ^13^Department of Cardiology, Hospital East-Limburg, Genk, Belgium; ^14^Department of Cardiovascular Sciences, University Hospitals Leuven, Leuven, Belgium; ^15^Caring Futures Institute, College of Nursing and Health Sciences, Flinders University, Adelaide, SA, Australia; ^16^Center for Heart Rhythm Disorders, University of Adelaide and Royal Adelaide Hospital, Adelaide, SA, Australia; ^17^Department of Biomedical Sciences, Faculty of Health and Medical Sciences, University of Copenhagen, Copenhagen, Denmark

**Keywords:** atrial fibrillation, mobile health, photoplethysmography, risk factors, thromboembolic risk

## Abstract

**Introduction:**

The TeleCheck-AF approach is an on-demand mobile health (mHealth) infrastructure incorporating mobile app-based heart rate and rhythm monitoring through teleconsultation. We evaluated feasibility and accuracy of self-reported mHealth-based AF risk factors and CHA_2_DS_2_-VASc-score in atrial fibrillation (AF) patients managed within this approach.

**Materials and Methods:**

Consecutive patients from eight international TeleCheck-AF centers were asked to complete an app-based 10-item questionnaire related to risk factors, associated conditions and CHA_2_DS_2_-VASc-score components. Patient's medical history was retrieved from electronic health records (EHR).

**Results:**

Among 994 patients, 954 (96%) patients (38% female, median age 65 years) completed the questionnaire and were included in this analysis. The accuracy of self-reported assessment was highest for pacemaker and anticoagulation treatment and lowest for heart failure and arrhythmias. Patients who knew that AF increases the stroke risk, more often had a 100% or ≥80% correlation between EHR- and app-based results compared to those who did not know (27 vs. 14% or 84 vs. 77%, *P* = 0.001). Thromboembolic events were more often reported in app (vs. EHR) in all countries, whereas higher self-reported hypertension and anticoagulant treatment were observed in Germany and heart failure in the Netherlands. If the app-based questionnaire alone was used for clinical decision-making on anticoagulation initiation, 26% of patients would have been undertreated and 6.1%—overtreated.

**Conclusion:**

Self-reported mHealth-based assessment of AF risk factors is feasible. It shows high accuracy of pacemaker and anticoagulation treatment, nevertheless, displays limited accuracy for some of the CHA_2_DS_2_-VASc-score components. Direct health care professional assessment of risk factors remains indispensable to ensure high quality clinical-decision making.

## Introduction

According to the current European Society of Cardiology (ESC) guidelines (1) for the diagnosis and management of atrial fibrillation (AF), treatment of AF incorporates heart rate or rhythm control, stroke prevention with appropriate anticoagulation therapy, and management of comorbidities, risk factors or lifestyle modification. The presence and combination of specific risk factors may trigger the prescription and frequent adjustment of medical therapies, e.g., anticoagulation, to prevent stroke, based on the CHA_2_DS_2_-VASc-score.

Traditionally, individual risk factors are assessed by structured face-to-face history taking during outpatient visits. During the coronavirus disease 2019 (COVID-19) pandemic, scheduled face-to-face outpatient consultations were frequently converted into teleconsultations (2). To support AF management through teleconsultations, a new mobile health (mHealth) approach was made available to several European AF centers within the large TeleCheck-AF project. This mHealth approach incorporated teleconsultations coupled with remote on-demand photoplethysmography (PPG)-based heart rate and rhythm monitoring (FibriCheck®) (3–6). Within the TeleCheck-AF project, patients were invited to fill in a 10-item questionnaire via the mobile phone app focusing on AF risk factors required to guide comprehensive AF management and estimate thromboembolic risk by the CHA_2_DS_2_-VASc-score. Although app-based questionnaires have been used previously in mHealth infrastructures (7, 8), the accuracy of self-reported data collected with a mobile app compared to clinical health records and possible consequences for clinical decision-making on the initiation of anticoagulation has not been investigated, yet.

Within the TeleCheck-AF project, we evaluated the feasibility and accuracy of a remote mobile app-based self-reported assessment of AF risk factors and CHA_2_DS_2_-VASc-score.

## Materials and Methods

### Project Design

The TeleCheck-AF project has been previously described in more detail (4). In brief, TeleCheck-AF is an international, multicenter on-demand mHealth infrastructure, initially dedicated to allowing the continuity of comprehensive AF management and to support integrated care through teleconsultation during the COVID-19 pandemic. It involves a structured teleconsultation (“Tele”) preceded by an app-based on-demand heart rate, rhythm, and symptom monitoring infrastructure (“Check”) to guarantee comprehensive AF management (“AF”). The retrospective data collection from the participating TeleCheck-AF centers was conducted in accordance with the Declaration of Helsinki and was approved by the local ethics committees of the participating centers.

### Patient Population

From April 2020 to April 2021, patients (≥18 years) scheduled for teleconsultation in 40 European AF outpatient clinics were managed within the TeleCheck-AF project. Individuals were eligible if they had a smartphone and were able to operate the remote on-demand heart rate, rhythm, and symptom monitoring mobile phone application system after instructions. A subgroup of these 40 centers participated in the retrospective analysis. Eight centers with the highest contribution in patient recruitment (recruited at least 25 patients) were included in this specific app-based AF risk factor assessment analysis (Maastricht University Medical Center+, Maastricht, the Netherlands; Radboud University Medical Center, Nijmegen, the Netherlands; Rijnstate, Arnhem, the Netherlands; Hannover Heart Rhythm Center, Hannover, Germany; University Hospital Cologne, Cologne, Germany; Medical University of Graz, Graz, Austria; Liverpool Heart and Chest Hospital, Liverpool, United Kingdom; Medical University of Warsaw, Warsaw, Poland).

### Project Procedures

At least 1 week prior to a scheduled (tele)consultation appointment, patients were provided with a mHealth prescription in the form of a temporary QR code and short instruction for the Conformité Européenne (CE)-marked app-based heart rate, rhythm, and symptom monitoring (FibriCheck, Qompium, Hasselt, Belgium) using PPG technology through the built-in camera of a mobile phone (4). Patients were instructed to record a 60-s PPG measurement and specify their symptoms, if any, three times daily and in case of symptoms for 7 consecutive days prior to their teleconsultation. Once the first measurement was performed, patients received a separate automatic app notification to complete a short mobile phone app-based 10-item questionnaire with closed-ended questions (yes or no) provided in different languages related to patient-reported AF risk factors presented in Supplementary Table 1. A reminder to complete the questionnaire automatically popped up after the following four app-based heart rate, rhythm, and symptom recordings (five times in total).

### Data Collection

The results of the questionnaire were collected in the FibriCheck cloud, an CE marked and secured online database, only accessible to authorized physicians, and afterwards exported for each center participating in the retrospective per-patient analysis.

A standardized electronic case record form was provided to all centers participating in the retrospective per-patient analysis of the TeleCheck-AF population. Baseline clinical characteristics (demographics and medical history) were retrieved from patients' electronic health records (EHR) at time of start app-based heart rate and rhythm monitoring. Each patient-reported app-based AF risk factor was compared with the corresponding EHR-based risk factor information, available in Supplementary Table 1. This process was blinded, as responsible physicians were not aware of the patient‘s response regarding the mHealth questionnaire.

Using the app-based AF risk factor information and EHR-based AF risk factor information, we calculated the app-based and EHR-based CHA_2_DS_2_-VASc-score, respectively. The potential risk for OAC undertreatment was defined as the number of patients that would not have been treated with appropriate anticoagulation if only the app-based risk factor questionnaire would have been used [patients with app-based CHA_2_DS_2_-VASc-score 0 (male), 1 (female) and EHR-based CHA_2_DS_2_-VASc-score ≥ 1 (male), ≥2 (female)] according to current ESC guidelines (1). The potential risk for OAC overtreatment was defined as the number of patients that would have been prescribed with anticoagulants without meeting indication criteria, if only the app-based risk factor questionnaire would have been used [patients with app-based CHA_2_DS_2_-VASc-score ≥1 (male), ≥2 (female) and EHR-based CHA_2_DS_2_-VASc-score 0 (male), 1 (female)].

### Statistical Analysis

All continuous variables were pretested for normal distribution using the Shapiro-Wilk test and assessed as non-parametric variables therefore presented as median (interquartile range [IQR]) and categorical variables as numbers (*n*) with percentages (%). Differences in continuous parameters were compared using non-parametric Wilcoxon signed-rank test or Mann-Whitney U test as applicable. For the comparison of categorical data, the McNemar's test or Chi-square test was used. For sensitivity and specificity comparison between participating countries, the McNemar's test was used. To determine predictors of app- and EHR agreement, multiple logistic regression analysis, using the stepwise backward procedure (with α level of 0.05) was performed, including all variables that reached significance in univariate analysis with continuous variables (age) assessed every 10 units (Supplementary Table 2). Finally, accuracy of app-based AF risk factor assessment was estimated by receiver operating characteristic (ROC) analysis, reporting sensitivity and specificity. Statistical significance was assumed at a 5% level. For database management and statistical analysis, IBM SPSS Version 25 (IBM Corporation, Somers, New York, USA) was used.

## Results

In eight of the most active TeleCheck-AF centers, 994 consecutive AF patients were available in the database. Out of these patients, 954 (96%) patients (363 female, age 65 [57–71] years) completed the mobile app-based 10-item questionnaire and were included in this analysis. No statistically significant difference was observed between patients who completed the questionnaire compared to those who did not complete it regarding baseline characteristics, except older age (65 years [57–71] vs.61 years [52–69], *P* = 0.046) (Supplementary Table 3).

### Agreement Between EHR and App-Based Parameters

The agreement between the mobile app-based 10-item questionnaire and the EHR is presented in Table 1. There were no statistically significant differences between EHR and app-based reported sex and age. Patients more often reported having a pacemaker in the mobile app (4.1 vs. 2.6% in EHR, *P* = 0.001). Arrhythmias (89.2 vs. 97.5%, *P* < 0.001), and in particular AF (69.3 vs. 90.2%, *P* < 0.001) were less often reported, whereas heart failure was more frequently reported (24.0 vs. 14.3%, *P* < 0.001) in the mobile app-based questionnaire compared to the EHR. Vascular disease was reported in 13.5% of patients in the mobile app, while vascular disease was mentioned by 15.7% of patients in the EHR (*P* = 0.057). There was a significant difference in the number of patients who had a medical history of TIA and/or CVA in the mobile app-based questionnaire compared to the EHR (25.9 vs. 8.9%, *P* < 0.001). A total of 274 (29.3%) patients reported hypertension in the mobile app-based questionnaire and as much as 461 (49.3%) patients had a diagnosis of hypertension in EHR (*P* < 0.001). The number of patients with diabetes mellitus was similar in the mobile app-based questionnaire and EHR (11.8 vs. 9.9%, *P* = 0.097). Anticoagulation treatment was similarly reported in both app and EHR (79.8 vs. 80.3%, *P* = 0.649). Overall, the sensitivity and specificity of the mobile app-based assessment was highest for pacemaker therapy and anticoagulant treatment, and lowest for vascular disease or heart attacks and arrhythmias. Noteworthy, arrhythmias including AF were not only less often reported but also more often inappropriately reported resulting in the lowest specificity (Table 2).

**Table 1:** Demographics and 10-item questionnaire compared to electronic health record-based results.

**App-based question**	**App-based results**	**EHR-based results**	***P*-value**
**Demographics**			
Female sex	369 (38.7%)	363 (38.1%)	0.210
Age (years), median [IQR]	65 [57–71]; *n = 895*	65 [57–71]; *n = 895*	0.213
**Questionnaire parameters**			
Did you know atrial fibrillation increases the risk of stroke?	630 (66.1%); *n = 953*	NA	NA
Do you have a pacemaker?	38 (4.1%); *n = 932*	24 (2.6%); *n = 932*	0.001
Were you ever diagnosed with cardiac arrhythmias?	828 (89.2%); *n = 928*	905 (97.5%); *n = 928*	<0.001
Are you (or were you before) diagnosed with or treated for atrial fibrillation or AF?	644 (69.3%); *n = 929*	838 (90.2%); *n = 929*	<0.001
Are you (or were you before) treated for heart failure or pulmonary edema?	224 (24.0%); *n = 934*	134 (14.3%); *n = 934*	<0.001
Are you (or were you before) treated for vascular disease in your legs or aorta? Or did you ever suffer from a heart attack?	126 (13.5%); *n = 936*	147 (15.7%); *n = 936*	0.057
Did you ever suffer from thrombosis or a stroke, with or without serious consequences (CVA or TIA)?	242 (25.9%); *n = 935*	83 (8.9%); *n = 935*	<0.001
Are you (or were you before) treated for hypertension?	274 (29.3%); *n = 935*	461 (49.3%); *n = 935*	<0.001
Are you (or were you before) treated for diabetes?	110 (11.8%); *n = 936*	93 (9.9%); *n = 936*	0.097
Do you take anticoagulants?	743 (79.8%); *n = 931*	748 (80.3%); *n = 931*	0.649
**Thromboembolic risk**			
CHA_2_DS_2_-VASc-score 0 (if male), 1 (if female)	204 (23.9%); *n = 853*	197 (23.1%); *n = 853*	0.468
CHA_2_DS_2_-VASc-score 1 (if male), 2 (if female)	176 (20.6%)	220 (25.8%)	0.002
CHA_2_DS_2_-VASc-score ≥ 2 (if male), ≥3 (if female)	473 (55.5%)	436 (51.1%)	0.004

*AF, atrial fibrillation; CVA, cerebrovascular accident; EHR, electronic health record; IQR, interquartile range; NA, non-applicable; TIA, transient ischemic attack. Number provided after the semicolon indicates the total number of patients available for that variable*.

**Table 2:** Sensitivity and specificity of app-based with electronic health record-based results.

**App-based question**	**Sensitivity**	**Specificity**
Do you have a pacemaker?	0.958	0.983
Were you ever diagnosed with cardiac arrhythmias?	0.898	0.348
Are you (or were you before) diagnosed with or treated for atrial fibrillation or AF?	0.724	0.593
Are you (or were you before) treated for vascular disease in your legs or aorta? Or did you ever suffer from a heart attack?	0.403	0.787
Are you (or were you before) treated for heart failure or pulmonary edema?	0.551	0.943
Did you ever suffer from thrombosis or a stroke, with or without serious consequences (CVA or TIA)?	0.723	0.786
Are you (or were you before) treated for hypertension?	0.497	0.905
Are you (or were you before) treated for diabetes?	0.591	0.935
Do you take anticoagulants?	0.945	0.803

*Abbreviations: see Table 1. The heatmap scale reflects the highest agreement between app- and EHR-based results (green) and the lowest agreement (red)*.

#### Patients With vs. Without Overall Full Agreement

**Figure 1:**
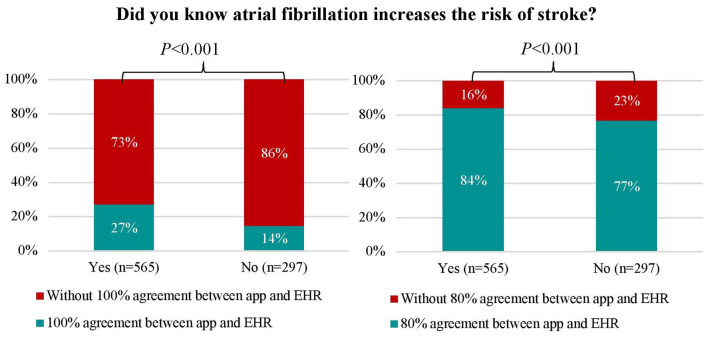
Comparison between patients with and without 100% and ≥80% agreement between electronic health record- and app-based results and patients' knowledge about atrial fibrillation as a risk for stroke. Abbreviations: see Table 1.

One-fifth of patients (*n* = 196 [22.7%]) completed the app-based questionnaire in 100% agreement with EHR. Those patients were younger (63 [56–70] vs.66 [57–72] years, *P* = 0.014), were more often diagnosed with AF (94.9 vs. 89.8%, *P* = 0.033) and more frequently treated with AF ablation therapy (63.3 vs. 38.5%, *P* < 0.001) to restore heart rhythm as compared to those whose responses on questionnaire were not in full agreement (Supplementary Table 2). Moreover, patients with 100% agreement had less comorbidities such as coronary artery disease, diabetes or hypertension. Additionally, they had lower thromboembolic risk and were less often treated with cardiovascular medications. Patients who reported awareness that AF increased the risk of stroke were more likely to have a 100% agreement (27 vs. 14%, *P* = 0.001) and ≥80% agreement (84 vs. 77%, *P* = 0.001) between EHR and app-based results compared to those who did not (Figure 1). Predictors for 100% app-EHR agreement were previous AF ablation therapy (odds ratio [OR] 2.40, 95% coincidence interval [CI] 1.64–3.51) and AF knowledge (OR 2.30, 95% CI 1.51–3.52), whereas coronary artery disease (OR 0.28, 95% CI 0.13–0.61), hypertension (OR 0.41, 95% CI 0.28–0.61) and beta-blocker therapy (OR 0.64, 95% CI 0.44–0.94) decreased this agreement (Supplementary Table 4).

#### Country Differences

In patients from all countries, hypertension was less frequently reported in the mobile app-based questionnaire compared to the EHR, while thromboembolic events such as TIA and/or CVA were more often reported. Some important country disparities between app- vs. EHR-based results were observed. Whereas, patients in Germany more often reported anticoagulant usage in the mobile app, Austrian patients reported such treatment less frequently. In addition, in contrary to German patients, Dutch patients more frequently declared having heart failure in app-based assessment (Supplementary Table 5).

#### Age Differences

Dividing patients into different age groups showed increasing tendency in anticoagulation usage and decreasing heart failure as well as vascular disease agreement between mobile app and EHR within patients aged between 30 and 80 years (Supplementary Figure 1).

### Assessment of Thromboembolic Risk and Anticoagulation

**Figure 2:**
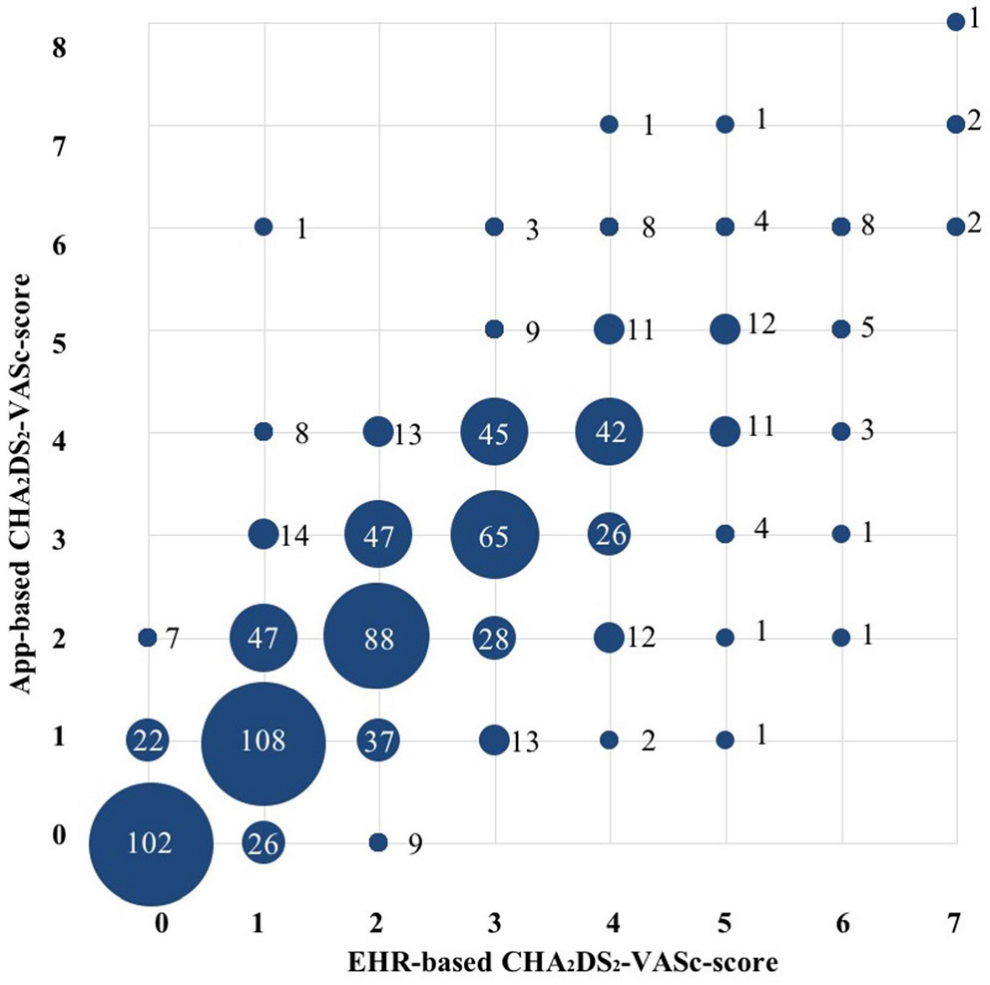
Comparison between electronic health record- and app-based CHA_2_DS_2_-VASc score (*n* = 853). Size of the circles represent the numbers of patients (also mentioned as numbers). Abbreviations: see Table 1.

**Figure 3:**
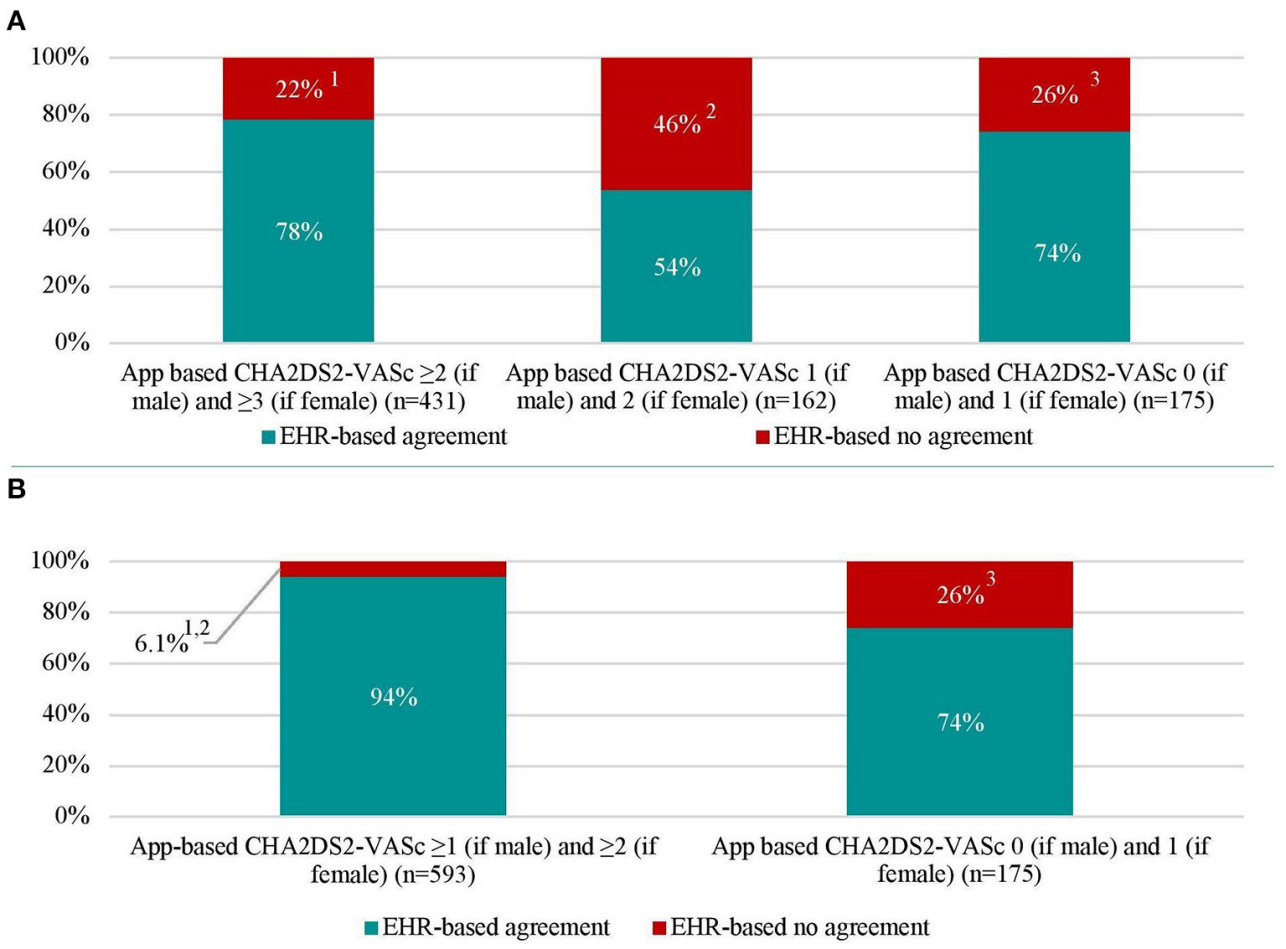
Thromboembolic (CHA_2_DS_2_-VASc) score in patients with atrial fibrillation based on electronic health record- and app-based results (*n* = 768). **(A)** represents recommended (App-based CHA_2_DS_2_-VASc ≥2 [if male] and ≥3 [if female]), to be considered (App-based CHA_2_DS_2_-VASc 1 [if male] and 2 [if female]), and not recommended (App-based CHA_2_DS_2_-VASc 0 [if male] and 1 [if female]) indications for oral anticoagulation with percentages of agreement and disagreement with electronic health record indications. **(B)** represents recommended and to be considered indications for oral anticoagulation were merged. Abbreviations: see Table 1.^1^EHR-based CHA_2_DS_2_-VASc 0 (if male) and 1 (if female) in 2.8% of patients, CHA_2_DS_2_-VASc 1 (if male) and 2 (if female) in 19% of patients. ^2^EHR-based CHA_2_DS_2_-VASc 0 (if male) and 1 (if female) in 15% of patients, CHA_2_DS_2_-VASc ≥2 (if male) and ≥3 (if female) in 31% of patients. ^3^EHR-based CHA_2_DS_2_-VASc ≥2 (if male) and ≥3 (if female) in 5.1% of patients, CHA_2_DS_2_-VASc 1 (if male) and 2 (if female) in 21% of patients.

CHA_2_DS_2_-VASc-scores were determined based on information derived from the mobile app and by information derived from the EHR. Compared to the CHA_2_DS_2_-VASc-score derived from data in the EHR, the mobile app-based assessment of the CHA_2_DS_2_-VASc-score identified a lower proportion of patients with a high thromboembolic risk and CHA_2_DS_2_-VASc-score ≥ 2 (if male), ≥3 (if female) (51.1 vs. 55.5%, *P* = 0.004) (Table 1 and Figure 2). Compared to the results from the EHR, the app-based assessment would have resulted in a different indications for OAC in one-fifth (22%) of patients with EHR-based CHA_2_DS_2_-VASc-score ≥2 (if male) and ≥3 (if female), half (46%) of patients with EHR-based CHA_2_DS_2_-VASc-score 1 (if male) and 2 (if female) and quarter (26%) of patients with EHR-based CHA_2_DS_2_-VASc-score 0 (if male) and 1 (if female) (Figure 3A). Compared to the CHA_2_DS_2_-VASc-score derived from data in the EHR, the app-based assessment of the CHA_2_DS_2_-VASc-score would have resulted in a different indications for OAC in 6.1% of patients with EHR-based CHA_2_DS_2_-VASc-score ≥1 (if male) and ≥2 (if female) and 26% of patients with EHR-based CHA_2_DS_2_-VASc-score 0 (if male) and 1 (if female) (Figure 3B). The proportion of patients with a CHA_2_DS_2_-VASc-score ≥1 (if male) and ≥2 (if female) based on the mobile app and the EHR was comparable (Supplementary Figure 2).

## Discussion

Surveys for AF risk factor assessment have been used in previous mHealth studies (7, 9–11). To the best of our knowledge, the present analysis of the real-world European mHealth TeleCheck-AF project conducted in numerous Telehealth-AF centers is the first assessing and validating the accuracy of remote self-reported AF risk factors and CHA_2_DS_2_-VASc-scores by patients, based on an app-based 10-item questionnaire in comparison with EHR data. Although blood pressure and physical activity data (12) can be directly incorporated into mobile apps by immediate data transfer from the measurement device, some other AF risk factors are filled in by patients and herein, we present the first study on accuracy of patient self-reported risk factor documentation.

We demonstrated that collection of patient self-reported AF risk factors by an app-based 10-item questionnaire is feasible. In a real-world setting within the TeleCheck-AF project, most patients completed the app-based questionnaire. Within this physician-initiated and patient-centered setting, all patients were provided a standard instruction to guide them through the installation and activation process of the app (4). Additionally, after installation of the app, pop-up messages were provided to remind patients to complete the questionnaire. The high completion rate of >90% demonstrates that a reminder-based questionnaire with a limited number of closed-ended questions is feasible making it an important tool for further digital studies. We found that older patients were more concordant in completing the app-based questionnaire. Moreover, compared to younger patients, these patients showed a higher agreement between app-based and EHR-based assessment of anticoagulation usage but lower agreement between app-based and EHR-based heart failure assessment. This suggests that age should not be a limitation for innovative solutions such as mHealth questionnaires. However, other factors such as lower health literacy, lower education and lower income, which was not specifically determined in TeleCheck-AF, may represent barriers for digital health usage and mHealth equity (13).

To determine the accuracy of app-based risk factors and CHA_2_DS_2_-VASc-score, we compared the information provided by patients via the app with the patient characteristics retrieved from the EHR completed by the treating physician and used to decide on patient management and treatment in the respective outpatient clinics of the participating TeleCheck-AF centers. Despite an acceptable accuracy of app-based AF risk factor assessment compared to EHR, there are still differences between mobile app and EHR. Possibly, the formulation and wording of questions enclosed in the 10-item questionnaire even in countries with same language (AF named as both, “voorkamerfibrilleren” and “boezemfibrilleren”) may explain some of the discrepancy observed (14). Furthermore, as TeleCheck-AF is an international mHealth project, language/country-specific differences in app-based questionnaire translations may also play a role in the differences between mobile app-based and EHR-based risk factor assessment. The difference between countries could also be explained by the different settings in which the TeleCheck-AF protocol was used in these countries (for example in Germany more often used in for pulmonary vein isolation follow up). Accordingly, Germany and Austria, which share German as a common language, document similar pattern of accuracy of app-based and EHR-based results. Likewise, in the Netherlands, a particularly high accuracy was observed, which may reflect the effect of more intense patient education in the dedicated AF outpatient clinics, which was not present in other countries participating in TeleCheck-AF. Whether better patient instruction and easier language use may improve the accuracy of app-based AF risk factor assessment warrants further studies. In general, a direct health care professional-patient contact, either as face-to-face consultation or teleconsultation, to critically check patient self-reported app-based statements regarding their medical-history and risk factors remains indispensable.

Differences between self-reported app-based AF risk factors and the EHR-based risk factors may support the treating health care provider to identify gaps in knowledge and awareness of the patients about their own risk factors. In a recent meta-analysis including 21 studies that assessed AF patients' knowledge about their medications and condition, the main AF-related knowledge gap and misconception was the fact that AF can be asymptomatic and can predispose to heart failure (15). This is in line with our results where patients underreported arrhythmias and overreported heart failure in the app-based questionnaire. Incorporating this information on possible knowledge gaps of our patients in traditional face-to-face consultations or teleconsultations can help to guide a personalized patient education. There is a growing number of mobile applications, educational platforms and websites (www.afibmatters.org) dedicated to improve patients' knowledge about AF (16) and compliance for treatment with anticoagulation. Based on our study, patient knowledge about AF as a risk factor for stroke was independently associated with higher agreement between EHR and app-based results. This adds to the result of recent studies suggesting, that a better knowledge about AF and associated treatment options increases the acceptance of adverse events associated with treatment and disease (17), anticoagulation adherence (18), symptom management and quality of life (19).

In addition to the above discussed limited accuracy of some of the app-based risk factors and the app-derived CHA_2_DS_2_-VASc-score, a purely digital assessment of AF patients does not incorporate factors such as frailty, kidney function and potential bleeding risk, which also need to be considered for the initiation of OAC treatment. In TeleCheck-AF, without considering clinical OAC contraindications and OAC indications other than AF, 26% of patients would be exposed to a potential risk for OAC undertreatment and 6% of patients to a potential risk for OAC overtreatment if only the app-based risk factor questionnaire would have been used for the clinical decision on the initiation of OAC (20). Whether this would be acceptable for the initiation of OAC in a purely digital AF management setting or whether the results could be used for future digital trials to describe patient characteristics needs to be further discussed with all involved stakeholders, including patients. Noteworthy, proper risk factor (CHA_2_DS_2_-VASC score) assessment is crucial in AF screening to identify high thromboembolic risk population.

In TeleCheck-AF, we used a 7 day on-demand mHealth approach. The completion of the 10-item questionnaire was just a spot assessment of the risk factors. However, risk differs due to individual temporally dynamic risk factors and may change over time. Therefore, close patient monitoring may make sense to regularly re-evaluate burden of AF as well as current risk factors (21, 22). App-based risk factor monitoring has potential for longitudinal risk factor assessment to evaluate treatment response and the development of new risk factors early. Including the possibility for frequent re-assessment of risk analysis over time by mHealth apps may allow future longitudinal analyses and assessments of risk factors which could be used to detect deterioration of risk factors at an early time point. Possibly, a structured longitudinal re-evaluation of risk scores may result in a better guideline adherence over time and guide individualized risk factor management programs. Therefore, the ideal setting may be longitudinal app-based questionnaire validated by physicians with the help of patient records during the teleconsultation.

### Limitations

Our study has several limitations. Firstly, there may be selection bias, as it includes only patients who were willing to use the mobile app in this real-life setting. Therefore, there should be caution in generalizing our findings to all patients with AF, especially living in non-wealthy countries. Secondly, due to the retrospective, observational character of this study, we were not able to determine the causal relationship between patient characteristics and completion of the 10-item questionnaire as well as the 100% agreement between mobile app and EHR. Thirdly, definitions of CHA_2_DS_2_-VASc-score components were fairly differently defined in app and EHR. Vascular disease was defined as peripheral artery disease or myocardial infarction in the app, but in the EHR, percutaneous coronary intervention and coronary artery bypass graft were included as well. In addition, hypertension in app was based on medication, although some hypertensive drugs such as angiotensin converting enzyme inhibitors may be given for other indications. This would have influenced the results, and these factors (vascular disease and hypertension) were also the components that varied the most. Finally, the timing of mobile app usage during the course of AF may have influenced app-based patient's knowledge concerning AF as newly diagnosed AF patients may be less aware of their disease than after a few months and few visits to the physician.

## Conclusion

App-based AF risk factor assessment is feasible. It shows high accuracy of pacemaker and anticoagulation treatment assessment, but limited accuracy for the assessment of some of the traditional AF risk factors as components of the CHA_2_DS_2_-VASc-score. As such, a direct doctor-patient contact remains indispensable to maintain high quality clinical-decision making, especially to prevent over- or undertreatment with prescribed anticoagulation. Whether app-based risk factor assessment can be incorporated in personalized patient education and longitudinal guidance of risk factor modification programs requires future studies.

## Data Availability Statement

The data underlying this article will be shared on reasonable request to the corresponding author.

## Ethics Statement

The studies involving human participants were reviewed and approved by Medical Research Ethics Committee of Maastricht University Medical Center (METC2020-1337). Written informed consent for participation was not required for this study in accordance with the national legislation and the institutional requirements.

## Author Contributions

AH, MG, HH, MM, DD, and DL were responsible for conception and design of the study. AH, MG, and HH were part of the data analysis committee and drafted the manuscript. All authors contributed substantially to data acquisition, data interpretation, critical revising of the manuscript for important intellectual content, to the final approval of the version to be published, and agreed to be accountable for all aspects of the work.

## Funding

The mHealth infrastructure was provided for free within the TeleCheck-AF project by the MUMC+ and additionally supported by FibriCheck® and BiosenseWebster®. The funder was not involved in the study design, collection, analysis, interpretation of data, the writing of this article or the decision to submit it for publication.

## Conflict of Interest

DSc has received speaker honoraria and/or travel grants from Bayer, Biosense Webster, Biotronik, BMS/Pfizer, Boehringer Ingelheim, Boston Scientific, Daiichi Sankyo, Medtronic, Zoll Medical, as well as research grants from Biosense Webster, Boston Scientific, and Zoll Medical. KV consultancy agreement with Boston, Medtronic, Biosense Webtster, Philips, and Abbott, Received educational grants from Medtronic, Abbott and Biosense Webster. JH declares that Flinders University has received speaker honoraria on his behalf from Biotronik. MM has received speaker honoraria and/or travel grants from Abbott, AOP Orphan, Bayer, Biotronik, Boston Scientific, Daiichi Sankyo, Pfizer, Zoll. DD has received speaker honoraria and/or travel grants from Abbott, Astra Zeneca, Bayer, Biotronik, Boehringer Ingelheim, Boston Scientific, Medtronic, Pfizer, Zoll. NK has received research grants from the Swiss National Science Foundation P400PM-194477, Gottfried und Julia Bangerter-Rhyner-Stiftung. The author acknowledges funding received from the European Society of Cardiology in form of an ESC Training Grant. The remaining authors declare that the research was conducted in the absence of any commercial or financial relationships that could be construed as a potential conflict of interest.

## Publisher's Note

All claims expressed in this article are solely those of the authors and do not necessarily represent those of their affiliated organizations, or those of the publisher, the editors and the reviewers. Any product that may be evaluated in this article, or claim that may be made by its manufacturer, is not guaranteed or endorsed by the publisher.
