# Supplementary material for: Self-Reported Mobile Health-Based Risk Factor and CHA2DS2-VASc-Score Assessment in Patients With Atrial Fibrillation: TeleCheck-AF Results
Source: Front Cardiovasc Med. 2022 Jan 19;8:757587. doi: 10.3389/fcvm.2021.757587 (PMC8809453; doi:10.3389/fcvm.2021.757587)
Supplement: Supplementary file 1 [file Data_Sheet_1.pdf]

Supplemental material

Self-reported mobile health-based risk factor and CHA_2DS_2-VASc-score assessment in patients with atrial fibrillation: TeleCheck-AF results.

Astrid NL Hermans[1]*, Monika Gawalko[1-3]*, Henrike AK Hillmann[4]*, Afzal Sohaib[5,6], Rachel MJ van der Velden[1], Konstanze Betz[1], Dominique Verhaert[1,7], Daniel Scherr[1,8], Julia Meier[8], Arian Sultan[9], Daniel Steven[9], Elena Terentieva[9], Ron Pisters[10], Martin Hemels[7,10], Leonard Voorhout[10], Piotr Lodzinski[3], Bartosz Krzowski[3], Dhiraj Gupta[11], Nikola Kozhuharov[11,12], Henri Gruwez[13,14], Kevin Vernooy[1], Nikki AHA Pluymaekers[1], Jeroen M Hendriks[15,16], Martin Manninger[8+], David Duncker[4+], Dominik Linz[1,7,16,17+]; on behalf of the TeleCheck-AF investigators

Shared first author*   Shared last author[+]

[1] Department of Cardiology, Maastricht University Medical Center and Cardiovascular Research Institute Maastricht, Maastricht, The Netherlands.

[2] Institute of Pharmacology, West German Heart and Vascular Center, University Duisburg-Essen, Germany

[3] 1st Department of Cardiology, Medical University of Warsaw, Warsaw, Poland

[4] Hannover Heart Rhythm Center, Department of Cardiology and Angiology, Hannover Medical School, Hannover, Germany.

[5] Barts Heart Center, St Bartholomew's Hospital, London, United Kingdom

[6] Department of Cardiology, King George Hospital, Ilford, United Kingdom.

[7] Department of Cardiology, Radboud University Medical Center, Nijmegen, the Netherlands.

[8] Department of Cardiology, University Clinic of Medicine, Medical University of Graz, Graz, Austria

[9] Department of Electrophysiology, University of Cologne, Heart Center, Cologne, Germany.

[10] Department of Cardiology, Rijnstate Hospital, Arnhem, the Netherlands

[11] Liverpool Heart and Chest Hospital, Liverpool, United Kingdom

[12] Department of Cardiology and Cardiovascular Research Institute Basel (CRIB), University Hospital Basel, University of Basel, Switzerland

[13] Department of Cardiology, Hospital East-Limburg, Genk, Belgium

[14] Department of Cardiovascular Sciences, University Hospitals Leuven, Leuven, Belgium

[15] Caring Futures Institute, College of Nursing and Health Sciences, Flinders University, Adelaide, Australia.

[16] Center for Heart Rhythm Disorders, University of Adelaide and Royal Adelaide Hospital, Adelaide, Australia.

[17] Department of Biomedical Sciences, Faculty of Health and Medical Sciences, University of Copenhagen, Copenhagen, Denmark.

Address for Correspondence:

Dominik Linz, MD, PhD

Maastricht UMC+, Maastricht Heart+Vascular Center

Universiteitssingel 50, 6229 ER Maastricht, the Netherlands

E dominik.linz@mumc.nl

T +31(0)43-3875093 | M +31(0)6-123 99 182

Supplemental material

Table S1. 10-item questionnaire

App-based question in English	App-based question in Dutch	App-based question in German	App-based question in Polish	EHR-based variable definition
Did you know atrial fibrillation increases the risk of stroke?	Wist je dat voorkamerfibrillatie of boezemfibrillatie het risico op een beroerte verhoogt?	Wussten Sie, dass Vorhofflimmern das Risiko eines Schlaganfalls erhöht?	Czy wiesz, że migotanie przedsionków zwiększa ryzyko udaru?	NA
Do you have a pacemaker?	Heb je een pacemaker?	Haben Sie einen Herzschrittmacher?	Czy masz stymulator serca?	Pacemaker
Were you ever diagnosed with cardiac arrhythmias?	Werden er al eerder hartritmestoornisse n bij je vastgesteld?	Wurden bereits Herzrhythmusstörunge n bei Ihnen festgestellt?	Czy kiedykolwiek zdiagnozowano u Ciebie arytmie	AF, atrial flutter, ectopic beats (PACs and PVCs) and/or SVT
Are you (or were you before) diagnosed with or treated for atrial fibrillation or AF?	Ben je (of was je voorheen) gediagnosticeerd of behandeld voor voorkamerfibrillatie of VKF?	Sind (oder waren) Sie wegen Vorhofflimmern (VHF) in Behandlung oder wurde diese Diagnose bei Ihnen gestellt?	Czy zdiagnozowano lub leczono migotanie przedsionków?	AF
Are you (or were you before) treated for heart failure or pulmonary edema?	Ben je (of was je voorheen) in behandeling voor hartfalen of longoedeem?	Sind (oder waren) Sie wegen Herzversagen oder eines Lungenödems in Behandlung?	Czy jesteś (lub byłeś wcześniej) leczony z powodu niewydolności serca lub zatorowości płucnej?	PE and/or HFrEF (LVEF ≤ 40%), HFmEF (LVEF 41 – 49%), or HFpEF (LVEF ≥ 50%)
Are you (or were you before) treated for vascular disease in your legs or aorta? Or did you ever suffer from a heart attack?	Ben je (of was je voorheen) in behandeling voor vaatlijden (bv. aderverkalking) in de benen of in de aorta? Of heb je ooit al een hartinfarct gekregen?	Sind (oder waren) Sie wegen Gefäßleiden in den Beinen oder der Aorta in Behandlung? Oder hatten Sie einen Herzinfarkt?	Czy jesteś (lub byłeś wcześniej) leczony z powodu miażdżycy w kończynach dolnych lub aorcie? A może kiedykolwiek miałeś zawał serca?	MI, PCI/PTCA, CABG, peripheral vascular disease
Did you ever suffer from thrombosis or a stroke, with or without serious consequences (CVA or TIA)?	Heb je ooit al een trombose of beroerte gehad, met of zonder nasleep (CVA of TIA)?	Hatten Sie bereits eine Thrombose oder einen Schlaganfall, mit oder ohne Nachwirkungen (CVA oder TIA)?	Czy kiedykolwiek miałeś zakrzepicę, udar mózgu lub przemijający atak niedokrwienny mózgu?	CVA and/or TIA
Are you (or were you before) treated for hypertension?	Ben je (of was je voorheen) in behandeling voor een te hoge bloeddruk?	Sind (oder waren) Sie wegen Bluthochdruck in Behandlung?	Czy jesteś (lub byłeś wcześniej) leczony na nadciśnienie?	Hypertension and/or hypertensive medication (ACEI, ARB, MRA, CCB, diuretics)
Are you (or were you before) treated for diabetes?	Ben je (of was je voorheen) in behandeling voor diabetes?	Sind (oder waren) Sie wegen Diabetes in Behandlung?	Czy jesteś (lub byłeś wcześniej) leczony na cukrzycę?	DMI, DMII and/or glucose-lowering medication

Supplemental material

Do you take anticoagulants?	Neem je een bloedverdunner/anti coagalant?	Nehmen Sie Gerinnungshemmer?	Czy bierzesz antykoagulanty?	NOAC and/or VKA

Abbreviations: ACEI, angiotensin-converting-enzyme inhibitor; AF, atrial fibrillation; ARB, angiotensin receptor blocker; CABG, coronary artery bypass grafting; CCB, calcium channel blocker; CVA, cerebrovascular accident; DM, diabetes mellitus; EHR, electronic health record; LVEF, left ventricular ejection fraction; MI, myocardial infarction; MRA, mineralocorticoid receptor antagonist; NOAC, novel oral anticoagulant; PAC, premature atrial contraction; PCI, percutaneous coronary intervention; PTCA, percutaneous transluminal coronary angioplasty; PVC, premature ventricular contraction; SVT, supraventricular tachycardia; TIA, transient ischemic attack; VKA, vitamin K antagonist

Legend: EHR-based "arrhythmias" variable includes patients who were ever diagnosed with AF, atrial flutter, ectopic beats (PACs and PVCs), and/or supraventricular tachycardia. The EHR-based "heart failure/PE" variable is defined as all patients diagnosed with PE and/or HFrEF (LVEF $\leq$ 40%), HFmEF (LVEF 41 – 49%), or HFpEF (LVEF $\geq$ 50%). EHR-based "artery disease" variable incorporates patients who were diagnosed with coronary artery disease (myocardial infarction, PCI/PTCA, or CABG and/or peripheral artery disease. EHR-based "AF" variable includes patients who were ever diagnosed with AF. The EHR-based "TIA/CVA" variable is defined as patients diagnosed with CVA and/or TIA. Patients diagnosed with hypertension and/or treated with hypertensive medication comprise the EHR-based variable "hypertension" and patients diagnosed with DM type 1 or type 2, and/or treated with glucose-lowering medication comprise the EHR-based variable "diabetes". The EHR-based "anticoagulants" variable is defined as patients treated with NOAC and/or VKA.

Supplemental material

Table S2. Comparison between patients who had 100% agreement in app-based risk factors and electronic health record-based variables and who had not.

Variable	100% in agreement (n=196)	Not 100% in agreement (n=666)	P-value
**Demographics**			
Female sex	72 (36.7%)	249 (37.4%)	0.933
Age (years)	63 [56 – 70]	66 [57 – 72]	0.014
BMI (kg/m2)	26.2 [23.8 – 29.0]; *n=186*	27.2 [24.7 – 30.4]; *n=606*	0.002
**AF**			
AF	186 (94.9%)	598 (89.8%)	0.033
First detected AF	9 (4.8%); *n=186*	64 (10.7%); *n=598*	0.014
Previous cardioversion (electrical and/or pharmaceutical)	80 (40.8%)	301 (45.3%); *n=665*	0.288
Ablation therapy for AF	124 (63.3%)	256 (38.5%); *n=665*	<0.001
Other arrhythmias	76 (45.5%); *n=167*	266 (43.5%); *n=611*	0.661
AF knowledge	153 (78.1%)	412 (61.2%)	<0.001
**Cardiovascular diseases**			
Coronary artery disease	8 (4.1%)	114 (17%)	<0.001
Peripheral vascular disease	0 (0.0%)	14 (2.1%)	0.049
Diabetes mellitus	9 (4.6%)	74 (11.1%)	0.023
Hypertension	57 (29.1%)	361 (54.2%)	<0.001
Congestive heart failure	14 (7.1%)	102 (15.3%); *n=666*	0.003
Obesity (BMI ≥30kg/m2)	32 (17.2%); *n=186*	160 (26.3%); *n=608*	0.011
Hypercholesterolemia	53 (27.6%); *n=192*	216 (33.0%); *n=655*	0.186
Stroke/TIA/pulmonary embolism	14 (7.1%)	72 (10.8%)	0.174
Hemorrhagic events	1 (0.5%)	2 (0.3%)	0.539
Device therapy (PM/CRT/ICD)	3 (1.5%)	38 (5.7%)	0.013
**Non-cardiovascular diseases**			
Obstructive sleep apnea syndrome	17 (13.8%); *n=123*	58 (12.0%); *n=484*	0.848
Chronic obstructive pulmonary disease	3 (1.5%)	47 (7.1%)	0.003
Chronic kidney disease	5 (2.6%)	34 (5.1%)	0.170
**Thromboembolic risk**			
CHA_2DS_2-VASc score 0 (if male), 1 (if female)	76 (39.6%); *n=192*	118 (18.2%); *n=649*	<0.001
CHA_2DS_2-VASc score 1 (if male), 2 (if female)	53 (27.6%); *n=192*	164 (25.3%); *n=649*	0.512
CHA_2DS_2-VASc score ≥ 2 (if male), ≥3 (if female)	63 (33%); *n=192*	367 (56.6%); *n=649*	<0.001
**Medications**			
Oral anticoagulants	141 (71.9%)	562 (84.3%)	<0.001
Antiplatelet drugs	2 (1.0%)	26 (3.9%)	0.063
Beta-blockers	87 (44.4%)	401 (60.2%)	<0.001
Antiarrhythmic drugs	70 (35.7%)	222 (33.3%)	0.546
Diuretics	32 (16.3%)	156 (23.4%)	0.039
Dihydropyridine-CCB	15 (7.7%)	97 (14.6%)	0.011
Non-dihydropyridine-CCB	6 (3.1%)	38 (5.7%)	0.195
ACEI	18 (9.2%)	136 (20.4%)	<0.001
ARB	33 (16.8%)	161 (24.2%)	0.032
MRA	6 (3.1%)	35 (5.3%)	0.254
Digoxin	7 (3.6%)	59 (8.9%)	0.014

Supplemental material

Number provided after the semicolon indicates the total number of patients available for that variable.

Abbreviations: AF, atrial fibrillation; ACEI; angiotensin-converting enzyme inhibitor; ARB, angiotensin II receptor blocker; BMI, body mass index; CCB, calcium channel blocker; CRT, cardiac resynchronization therapy; ICD, implantable cardioverter-defibrillator; MRA, mineralocorticoid receptor antagonist; PM. pacemaker; TIA, transient ischemic attack

Supplemental material

Table S3. Comparison between patients who completed and who did not complete the mobile app-based questionnaire.

Variable	Completed (n=954)	Not completed (n=40)	P-value
**Demographics**			
Female sex	363 (38.1%)	17 (42.5%)	0.570
Age (years)	65 [57 – 71]	61 [52 – 69]	0.046*
BMI (kg/m2)	27.1 [24.5 – 29.8]; *n=860*	27.4 [24.8 – 31.9]; *n=37*	0.395
**AF**			
AF	839 (90.2%); *n=930*	33 (84.6%); *n=39*	0.270
First detected AF	75 (9.0%); *n=836*	2 (6.1%); *n=33*	0.761
Previous cardioversion (electrical and/or pharmaceutical)	408 (42.8%); *n=953*	21 (52.5%)	0.255
Ablation therapy for AF	410 (43.0%); *n=953*	12 (30.0%)	0.103
Other arrhythmias	370 (43.8%); *n=844*	15 (42.9%); *n=35*	0.909
AF knowledge	630 (66.1%)	NA	NA
**Cardiovascular diseases**			
Coronary artery disease	138 (14.7%); *n=936*	4 (10.0%)	0.499
Peripheral vascular disease	16 (1.6%); *n=936*	0 (0.0%)	1.000
Diabetes mellitus	93 (9.9%); *n=936*	6 (15.0%)	0.284
Hypertension	461 (49.3%); *n=935*	24 (60.0%)	0.185
Congestive heart failure	134 (14.3%); *n=936*	7 (17.5%)	0.575
Obesity (BMI ≥30kg/m2)	206 (24.0%); *n=860*	14 (37.8%);*n=37*	0.055
Hypercholesterolemia	300 (32.6%); *n=919*	13 (32.5%)	0.985
Stroke/TIA/pulmonary embolism	91 (9.7%); *n=936*	5 (12.5%)	0.584
Hemorrhagic events	5 (0.5%); *n=937*	0 (0%)	1.000
Device therapy (PM/CRT/ICD)	45 (4.8%); *n=933*	3 (7.5%)	0.442
**Non-cardiovascular diseases**			
Obstructive sleep apnea syndrome	80 (11.8%); *n=676*	4 (12.9%); *n=31*	0.778
Chronic obstructive pulmonary disease	50 (5.3%); *n=937*	1 (2.5%)	0.717
Chronic kidney disease	46 (4.9%); *n=937*	2 (5.0%)	1.000
**Thromboembolic risk**			
CHA_2DS_2-VASc score 0 (if male), 1 (if female)	210 (23.0%); *n=912*	13 (33.3%); *n=39*	0.174
CHA_2DS_2-VASc score 1 (if male), 2 (if female)	235 (25.8%); *n=912*	10 (25.6%); *n=39*	1.000
CHA_2DS_2-VASc score ≥ 2 (if male), ≥3 (if female)	467 (51.2%); *n=912*	16 (41.0%); *n=39*	0.253
**Medications**			
Oral anticoagulants	748 (80.3%); *n=931*	30 (75.0%)	0.407
Antiplatelet drugs	34 (3.7%); *n=931*	2 (5.0%)	0.656
Beta-blockers	535 (57.5%); *n=930*	27 (67.5%)	0.211
Antiarrhythmic drugs	303 (32.6%); *n=930*	16 (40.0%)	0.328
Diuretics	204 (21.9%); *n=930*	10 (25.0%)	0.647
Dihydropyridine-CCB	117 (12.6%); *n=930*	5 (12.5%)	0.988
Non-dihydropyridine-CCB	53 (5.7%); *n=930*	3 (7.5%)	0.498
ACEI	171 (18.4%); *n=930*	9 (22.5%)	0.512
ARB	214 (23.0%); *n=930*	12 (30.0%)	0.306
MRA	49 (5.3%); *n=930*	4 (10.0%)	0.270

Digoxin	72 (7.7%); *n=930*	1 (2.5%)	0.356

Number provided after the semicolon indicates the total number of patients available for that variable.

Abbreviations: See **Table S2**

Supplemental material

Table S4. Predictors of 100% app-electronic health record agreement.

Parameter	OR	95% CI	*P*-value
Previous AF ablation therapy	2.40	1.64-3.51	<0.001
AF knowledge	2.30	1.51-3.52	<0.001
Coronary artery disease	0.28	0.13-0.61	<0.001
Hypertension	0.41	0.28-0.61	<0.001
Beta-blocker therapy	0.64	0.44-0.94	0.023

Abbreviations: AF, atrial fibrillation**;** CI, confidence interval; OR, odds ratio

Supplemental material

Table S5. Demographics and 10-item questionnaire compared to electronic health record-based results per country.

Variable	Electronic health record-based	App-based	P-value
**The Netherlands (n=630)**			
**Demographics**			
Female sex	249 (39.5%)	250 (39.7%)	1.000
Age (years)	67 [59 – 72]	67 [59 – 72]	0.050
**Questionnaire parameters**			
Did you know atrial fibrillation increases the risk of stroke?	NA	329 (52.3%); *n=629*	NA
Do you have a pacemaker?	14 (2.3%); *n=611*	19 (3.1%); *n=611*	0.063
Were you ever diagnosed with cardiac arrhythmias?	601 (98.7%); *n=609*	524 (86.0%); *n=609*	<0.001
Are you (or were you before) diagnosed with or treated for atrial fibrillation or AF?	557 (91.2%); *n=611*	352 (57.6%); *n=611*	<0.001
Are you (or were you before) treated for heart failure or pulmonary edema?	89 (14.6%); *n=611*	199 (32.6%); *n=611*	<0.001
Are you (or were you before) treated for vascular disease in your legs or aorta? Or did you ever suffer from a heart attack?	89 (14.5%); *n=613*	77 (12.6%); *n=613*	0.175
Did you ever suffer from thrombosis or a stroke, with or without serious consequences (CVA or TIA)?	68 (11.1%); *n=612*	86 (14.1%); *n=612*	0.015
Are you (or were you before) treated for hypertension?	288 (47.0%); *n=613*	246 (40.1%); *n=613*	<0.001
Are you (or were you before) treated for diabetes?	65 (10.6%); *n=613*	72 (11.7%); *n=613*	0.311
Do you take anticoagulants?	502 (82.2%); *n=611*	505 (82.7%); *n=611*	0.720
**Thromboembolic risk**			
CHA_2DS_2-VASc score 0 (if male), 1 (if female)	119 (19.7%); *n=603*	122 (20.2%); *n=603*	0.691
CHA_2DS_2-VASc score 1 (if male), 2 (if female)	160 (26.5%); *n=603*	144 (23.9%); *n=603*	0.176
CHA_2DS_2-VASc score ≥ 2 (if male), ≥3 (if female)	324 (53.7%); *n=603*	337 (55.9%); *n=603*	0.205
**Germany (n=191)**			
**Demographics**			
Female sex	71 (37.2%)	73 (38.2%)	0.625
Age (years)	65 [53 – 71]; *n=132*	64 [54 – 71]; *n=132*	0.937
**Questionnaire parameters**			
Did you know atrial fibrillation increases the risk of stroke?	NA	177 (92.7%)	NA
Do you have a pacemaker?	7 (3.7%); *n=190*	12 (6.3%); *n=190*	0.063
Were you ever diagnosed with cardiac arrhythmias?	174 (92.6%); *n=188*	181 (96.3%); *n=188*	0.143
Are you (or were you before) diagnosed with or treated for atrial fibrillation or AF?	156 (83.4%); *n=187*	167 (89.3%); *n=187*	0.013

Supplemental material

Are you (or were you before) treated for heart failure or pulmonary edema?	32 (16.8%); *n=190*	13 (6.8%); *n=190*	0.004
Are you (or were you before) treated for vascular disease in your legs or aorta? Or did you ever suffer from a heart attack?	41 (21.6%); *n=190*	37 (19.5%); *n=190*	0.585
Did you ever suffer from thrombosis or a stroke, with or without serious consequences (CVA or TIA)?	12 (6.3%); *n=190*	100 (52.6%); *n=190*	<0.001
Are you (or were you before) treated for hypertension?	105 (55.6%); *n=189*	20 (10.6%); *n=189*	<0.001
Are you (or were you before) treated for diabetes?	19 (10.0%); *n=190*	27 (14.2%); *n=190*	0.268
Do you take anticoagulants?	133 (70.4%); *n=189*	142 (75.1%); *n=189*	0.049
**Thromboembolic risk**			
CHA_2DS_2-VASc score 0 (if male), 1 (if female)	40 (30.8%); *n=130*	38 (29.2%); *n=130*	0.527
CHA_2DS_2-VASc score 1 (if male), 2 (if female)	21 (16.2%); *n=130*	13 (10.0%); *n=130*	0.088
CHA_2DS_2-VASc score ≥ 2 (if male), ≥3 (if female)	69 (79.8%); *n=130*	79 (60.1%); *n=130*	0.033
**Austria (n=79)**			
**Demographics**			
Female sex	24 (30.4%)	27 (34.2%)	0.453
Age (years)	58 [51 – 63]	58 [50 – 64]	0.670
**Questionnaire parameters**			
Did you know atrial fibrillation increases the risk of stroke?	NA	72 (91.1%)	NA
Do you have a pacemaker?	1 (1.3%); *n=77*	4 (5.2%); *n=77*	0.250
Were you ever diagnosed with cardiac arrhythmias?	76 (98.7%); *n=77*	71 (92.2%); *n=77*	0.125
Are you (or were you before) diagnosed with or treated for atrial fibrillation or AF?	72 (93.5%); *n=77*	72 (93.5%); *n=77*	1.000
Are you (or were you before) treated for heart failure or pulmonary edema?	7 (8.9%)	4 (5.1%)	0.508
Are you (or were you before) treated for vascular disease in your legs or aorta? Or did you ever suffer from a heart attack?	7 (8.9%)	4 (5.1%)	0.375
Did you ever suffer from thrombosis or a stroke, with or without serious consequences (CVA or TIA)?	2 (2.5%)	29 (36.7%)	<0.001
Are you (or were you before) treated for hypertension?	43 (54.4%)	1 (1.3%)	<0.001
Are you (or were you before) treated for diabetes?	4 (5.1%)	8 (10.1%)	0.344
Do you take anticoagulants?	72 (91.1%)	61 (77.2%)	0.027
**Thromboembolic risk**			
CHA_2DS_2-VASc score 0 (if male), 1 (if female)	23 (60.0%); *n=69*	30 (43.5%); *n=69*	0.090
CHA_2DS_2-VASc score 1 (if male), 2 (if female)	25 (36.2%) *n=69*	12 (17.4%); *n=69*	0.016

Supplemental material

CHA_2DS_2-VASc score ≥ 2 (if male), ≥3 (if female)	21 (30.4%); *n=69*	27 (39.1%); *n=69*	0.239
**Poland (n=30)**			
**Demographics**			
Female sex	13 (43.3%)	13 (43.3%)	1.000
Age (years)	60 [50 – 68]	60 [50 – 68]	0.317
**Questionnaire parameters**			
Did you know atrial fibrillation increases the risk of stroke?	NA	28 (93.3%)	NA
Do you have a pacemaker?	1 (3.3%)	1 (3.3%)	1.000
Were you ever diagnosed with cardiac arrhythmias?	30 (100.0%)	30 (100.0%)	1.000
Are you (or were you before) diagnosed with or treated for atrial fibrillation or AF?	30 (100.0%)	29 (96.7%)	1.000
Are you (or were you before) treated for heart failure or pulmonary edema?	2 (6.7%)	6 (20.0%)	0.219
Are you (or were you before) treated for vascular disease in your legs or aorta? Or did you ever suffer from a heart attack?	7 (23.3%)	5 (16.7%)	0.687
Did you ever suffer from thrombosis or a stroke, with or without serious consequences (CVA or TIA)?	0 (0.0%)	20 (66.7%)	<0.001
Are you (or were you before) treated for hypertension?	20 (66.7%)	5 (16.7%)	<0.001
Are you (or were you before) treated for diabetes?	3 (10.0%)	1 (3.3%)	0.625
Do you take anticoagulants?	29 (100.0%); *n=29*	23 (79.3%); *n=29*	0.008
**Thromboembolic risk**			
CHA_2DS_2-VASc score 0 (if male), 1 (if female)	7 (23.3%)	5 (16.7%)	0.778
CHA_2DS_2-VASc score 1 (if male), 2 (if female)	9 (30.0%)	4 (13.3%)	0.132
CHA_2DS_2-VASc score ≥ 2 (if male), ≥3 (if female)	14 (46.7%)	21 (70.0%)	0.035
**United Kingdom (n=24)**			
**Demographics**			
Female sex	6 (25.0%)	6 (25.0%)	1.000
Age (years)	64 [55 – 71]	64 [55 – 71]	0.317
**Questionnaire parameters**			
Did you know atrial fibrillation increases the risk of stroke?	NA	24 (100%)	NA
Do you have a pacemaker?	1 (4.2%)	2 (8.3%)	1.000
Were you ever diagnosed with cardiac arrhythmias?	24 (100.0%)	22 (91.7%)	0.687
Are you (or were you before) diagnosed with or treated for atrial fibrillation or AF?	23 (95.8%)	24 (100.0%)	1.000
Are you (or were you before) treated for heart failure or pulmonary edema?	4 (16.7%)	2 (8.3%)	0.687
Are you (or were you before) treated for vascular disease in your legs or aorta?	3 (12.5%)	3 (12.5%)	1.000

Supplemental material

Or did you ever suffer from a heart attack?			
Did you ever suffer from thrombosis or a stroke, with or without serious consequences (CVA or TIA)?	1 (4.2%)	7 (29.2%)	0.031
Are you (or were you before) treated for hypertension?	5 (20.8%)	2 (8.3%)	0.453
Are you (or were you before) treated for diabetes?	2 (8.3%)	2 (8.3%)	1.000
Do you take anticoagulants?	12 (52.2%); *n=23*	12 (52.2%); *n=23*	1.000
**Thromboembolic risk**			
CHA_2DS_2VASc-score 0 (if male), 1 (if female)	8 (38.1%); *n=21*	9 (42.9%); *n=21*	0.564
CHA_2DS_2VASc-score 1 (if male), 2 (if female)	5 (23.9%); *n=21*	3 (14.3%); *n=21*	0.414
CHA_2DS_2VASc-score ≥ 2 (if male), ≥3 (if female)	8 (38.1%); *n=21*	9 (42.9%); *n=21*	0.564

Legend: Number provided after the semicolon indicates the total number of patients available for that variable.

Abbreviations: See **Table S1**

Supplemental material

Figure S1. App-based questions in agreement with electronic health records depending on age group.

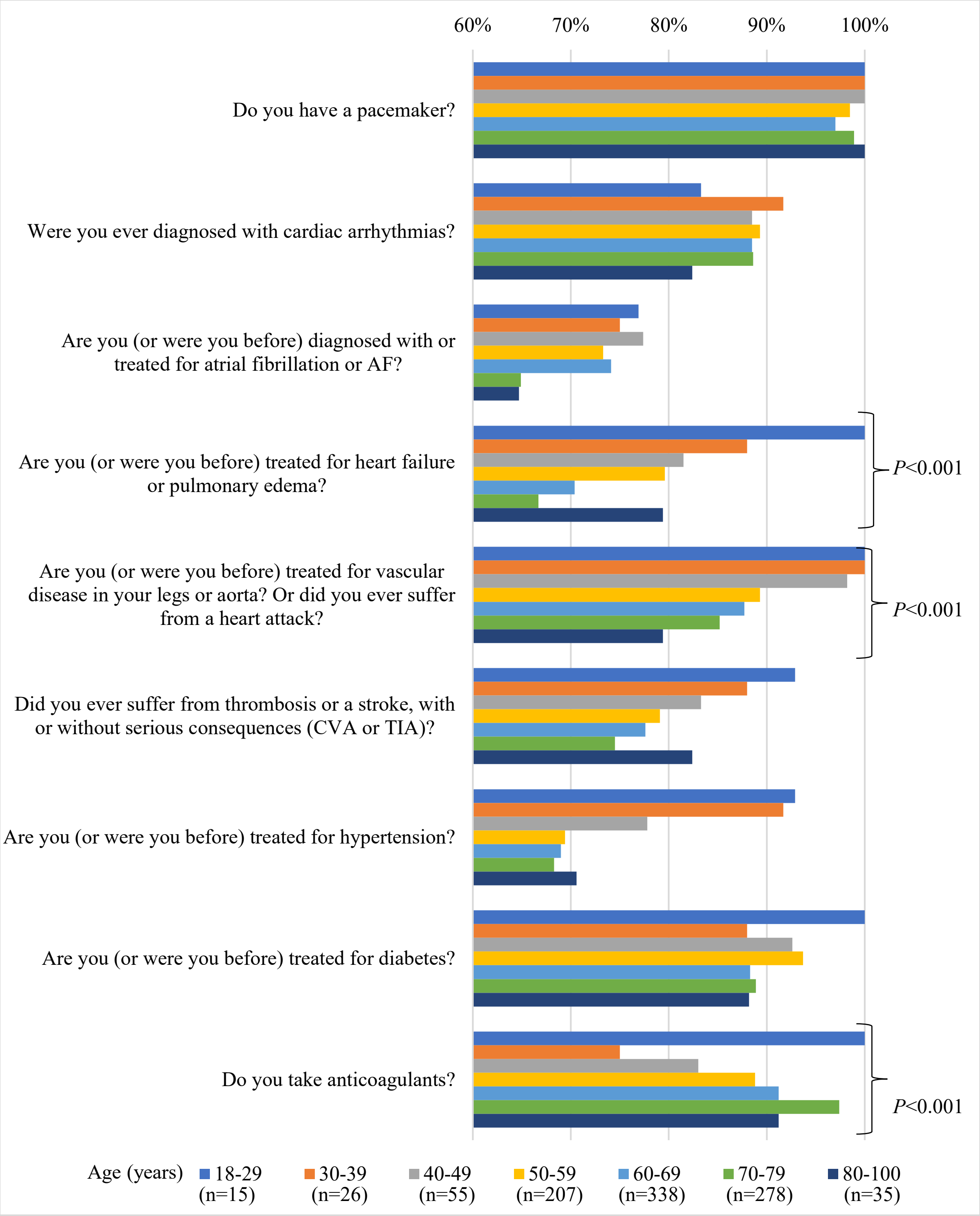

Abbreviations: See **Table S1**

Supplemental material

Figure S2. Accuracy and agreement of app-based vs electronic health record-based choice to oral anticoagulation in patients with atrial fibrillation (n=765[1]).

A. Accuracy of app- and electronic health-based choice to oral anticoagulation.

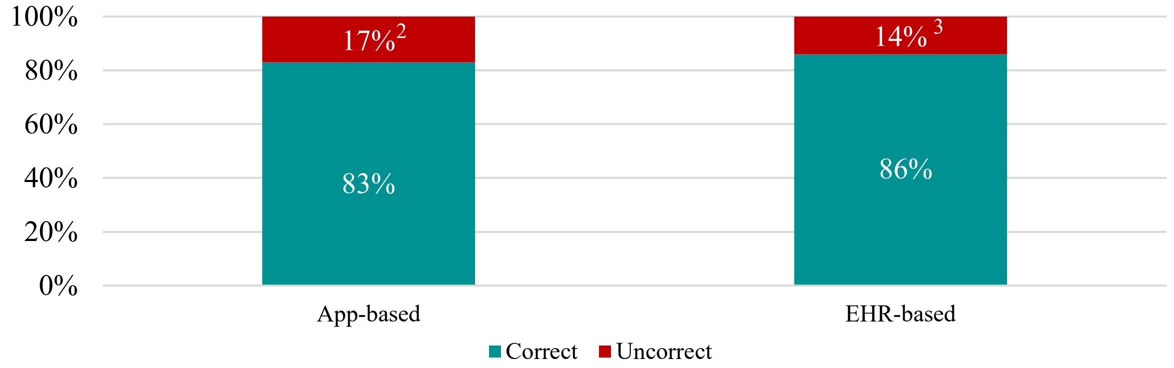

B. Agreement between app- and electronic health-based choice to oral anticoagulation.

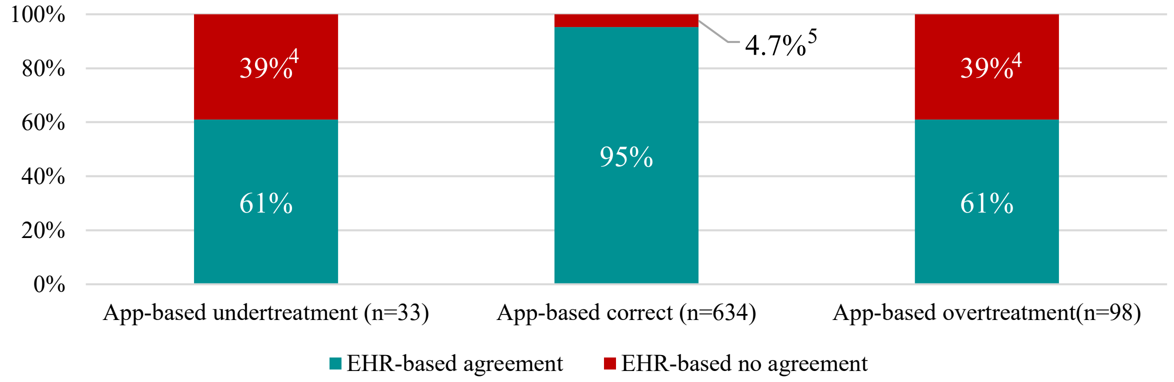

The accuracy of app-based and EHR-based choice to OAC should be taken with caution, as it's based only on thromboembolic score without consideration renal function, bleeding risk or valvular heart disease.

Abbreviations: EHR, electronic health record; OAC, oral anticoagulation

Legend:

[1] 3 patients with CHA_2DS_2-VASc ≥2 (if male) and ≥3 (if female), without information on anticoagulation

[2] App-based overtreatment in 13% of patients and undertreatment in 4.3% of patients

[3] EHR-based overtreatment in 11% of patients and undertreatment in 3.5% of patients

[4] EHR-based correct

[5] EHR-based overtreatment in 3.6% of patients and undertreatment in 1.1% of patients

App-based_correct: OAC treatment (based on EHR) in patients with CHA_2DS_2-VASc ≥1 (if male) and ≥2 (if female), based on app

EHR-based_correct: OAC treatment (based on EHR) in patients with CHA_2DS_2-VASc ≥1 (if male) and ≥2 (if female), based on EHR

Supplemental material

App-based_undertreatment: no OAC treatment (based on EHR) in patients with CHA_2DS_2-VASc ≥1 (if male) and ≥2 (if female) based on app

EHR-based_undertreatment: no OAC treatment (based on EHR) in patients with CHA_2DS_2-VASc ≥1 (if male) and ≥2 (if female) based on EHR

App-based_overtreatment: OAC treatment (based on EHR) in patients with CHA_2DS_2-VASc 0 (if male) and 1 (if female) based on app

EHR-based_overtreatment: OAC treatment (based on EHR) in patients with CHA_2DS_2-VASc 0 (if male) and 1 (if female) based on EHR
